# Attenuation of L-Type Ca^2+^ Channel Expression and Vasomotor Response in the Aorta with Age in Both Wistar-Kyoto and Spontaneously Hypertensive Rats

**DOI:** 10.1371/journal.pone.0088975

**Published:** 2014-02-12

**Authors:** Toshihiko Fukuda, Takahiro Kuroda, Miki Kono, Takahisa Miyamoto, Mitsuru Tanaka, Toshiro Matsui

**Affiliations:** The Department of Bioscience and Biotechnology, Faculty of Agriculture, Graduate School of Kyushu University, Hakozaki, Higashi-ku, Fukuoka, Japan; Medical College of Wisconsin, United States of America

## Abstract

Age-related vascular diseases are induced by vascular dysfunction, which involves changes in the vasomotor response. The voltage-dependent L-type calcium channel (VDCC) protein is involved in the regulation of vessel function (contraction/relaxation action). In the present study, we evaluated age-related vasomotor function and expression of the signal-related target proteins, including VDCC, using thoracic aorta from both 8- and 40-week old Wistar-Kyoto (WKY) and spontaneously hypertensive rats (SHR). In contraction experiments using aortic rings, vasomotor responses of both phenylephrine-induced contraction and acetylcholine-induced relaxation were significantly attenuated with age in SHR, whereas WKY did not lose activity with age. Contraction induced by angiotensin II was impaired only for the 40-week old SHR among all the rat groups tested, although enhanced AT1R/reduced AT2R expression with age was observed for both WKY and SHR. In contrast, a vasomotor responsiveness to Bay K 8644 (a VDCC agonist) at the initial contraction phase was significantly attenuated in both 40-week WKY and SHR with significant reduction of VDCC protein expression. The reduced VDCC expression in 40-week old rats significantly lowered the relaxation activity of VDCC blockers, such as verapamil and Trp-His, but did not affect that of nifedipine. Taken together, we provided the first evidence that aging caused a reduction of VDCC expression in rat aorta, irrespective of the rat strain, along with diminishment of the therapeutic potential of VDCC blockers.

## Introduction

It has been reported that aging greatly affects vessel tone and arterial stiffness [1], causing the onset of vascular-related diseases such as hypertension, diabetes mellitus and atherosclerosis, by arterial dysfunction of receptors, ion channels, and signal transduction pathways [2], [3]. So far, Touyz et al. [4] have demonstrated that aging augmented an expression of the pressor receptor, angiotensin II (Ang II) type I receptor (AT1R), while the depressor Ang II type II receptor (AT2R) was blunted in aged rat vessels. However, no studies have been reported regarding the relationship between age and expression of the voltage-dependent L-type Ca^2+^ channel (VDCC) in aorta. There are recent reports on its marked reduction in sinoatrial node [5] and in cerebral arteries [6] with age.

Regulation of the intracellular Ca^2+^ concentration ([Ca^2+^]_i_) is a beneficial initial therapeutic strategy for hypertension, since the Ca^2+^-related signaling cascade plays a role in vessel contraction mediated by myosin-light chain (MLC) phosphorylation [7]. In addition, Ishii et al. [8] reported an alternative therapeutic effect of blocking the cascade, in which a 10-week oral administration of nifedipine (10 mg/kg/day, a VDCC blocker) markedly suppressed the onset of atherosclerosis in apo E-deficient mice. Our previous study on the anti-atherosclerotic effect of the vasoactive di-peptide, Trp-His, which suppressed elevated [Ca^2+^]_i_ by binding to extracellular VDCC protein [9] and by inhibiting the phosphorylation of VDCC [10], also found inhibition of progressive atherosclerotic lesions in the mice [11]. Therefore, in order to improve vessel tone and/or prevent dysfunction with aging, it is important to clarify the relationship between age and vasomotor response.

Thus, in this study, we primarily investigated the effect of aging and/or hypertension on the vasomotor response (contraction/relaxation) using thoracic aorta from both 8- and 40-week old Wistar-Kyoto (WKY) and spontaneously hypertensive rats (SHR) as a function of vasomotor-related receptor and channel expression. Furthermore, we clarified whether compounds having VDCC blocking activity retained their relaxation potential in aged rat vessels.

## Experimental Procedures

### Drugs and Reagents

Phenylephrine (PE) and acetylcholine (ACh) were purchased from Wako Pure Chemical Ind. (Osaka, Japan). Ang II, PD123177, Bay K 8644, verapamil and both rabbit anti-AT1R and anti-AT2R primary antibodies were purchased from Sigma-Aldrich (St. Louis, MO, USA). Nifedipine was purchased from Nacalai Tesque (Kyoto, Japan). Trp-His was synthesized using an Fmoc-solid phase synthesis method, according to the manufacturer's instructions (Kokusan Chemicals, Tokyo, Japan). Rabbit anti-Cav1.2, the alpha-1c subunit VDCC, primary antibody was obtained from Alomone Labs (Jerusalem, Israel). Mouse anti-β-actin primary antibody was obtained from Applied Biological Materials Inc. (Richmond, BC, Canada). Horseradish peroxidase (HRP)-conjugated secondary antibodies and ECL detection reagents were obtained from GE Healthcare Biosciences (Piscataway, NJ, USA). Alexa 488-conjugated secondary antibody was obtained from Life Technologies (Carlsbad, CA, USA). All other chemicals were of analytical reagent grade and were used without further purification.

### Ethics Statement

All animal experiments were carried out under the Guidance for Animal Experiments in the Faculty of Agriculture in the Graduate Course of Kyushu University, and in accordance with Law (No. 105, 1973) and Notification (No. 6, 1980 of the Prime Minister's Office) of the Japanese Government. All experiments were reviewed and approved by the Animal Care and Use Committee of Kyushu University (Permit Number: A24-051).

### Animals and Preparation of Aortic Rings

Male 7-week-old and 39-week-old Wistar-Kyoto rats (WKY, WKY/NCrlCrij) and age-matched spontaneously hypertensive rats (SHR, SHR/NCrlCrij) were supplied by Charles River Japan (Kanagawa, Japan). The rats were acclimatized under laboratory condition (21±1°C; 55.5±5% humidity; 12 h dark/light cycle) for one week before the experiments, fed on a certificated basal diet in pellet form (MF diet, Oriental Yeast Co., Tokyo, Japan) and given water *ad libitum*. Preparation of thoracic aortic rings was performed as in our previous study [12]. Briefly, the thoracic aortae from euthanized rats were carefully excised and equilibrated for 45 min in physiological salt solution (PSS) buffer (pH 7.4) at 37°C. The PSS buffer had the following composition (in millimoles): NaCl 145, KCl 5, Na_2_HPO_4_ 1, CaCl_2_ 2.5, MgSO_4_ 0.5, glucose 10, and HEPES 5. After equilibration, the thoracic aortae were cleaned of adhering fat and connective tissue and were cut into rings 2 to 3 mm in length. The ring segments were each mounted between two stainless steel wires in a 5-mL organ bath filled with PSS buffer with 95% O_2_/5% CO_2_ gas. The rings were then progressively stretched to a preloaded tension of 2 g followed by an equilibration for another 45 min until stabilized. The vasomotor responses (isometric tension, in g) were measured by a force transducer (Micro Tissue Organ Bath, Model MTOB-1Z; Labo Support, Osaka, Japan) coupled to a data acquisition system (4-channel amplifier; EMKA Technologies, Paris, France). To verify the viability of the aortic rings, a 1 µM PE-contracted response with more than 0.25 g in isometric tension was confirmed before sample-induced relaxation experiments.

### Measurement of Vasomotor Response in Isolated Aortic Rings

Vasomotor responses in aortic rings from 8- and 40-week WKY and SHR were evaluated by either contraction or relaxation reagents. Aortic rings were primarily contracted with PE. Increased tension when the aortic rings were exposed to 1 µM PE was defined as a maximal contraction response of the aortic ring samples in these experiments. Subsequently, 10 µM ACh was added to the ring samples to obtain a maximal relaxation response. After washing the rings with PSS buffer, the rings were then subjected to either 1 µM Ang II or 1 µM Bay K 8644 stimulation experiment to obtain the contraction potential of the rings with each agonist. In Ang II-stimulation experiments, PD123177 (1 µM, an antagonist of AT2R [13]) was added 10 min before the addition of Ang II to avoid activation of relaxation signaling pathways via AT2R. Maximal tension after 1 µM Ang II addition to the rings was used for the evaluation of Ang II-induced contraction. Contraction by Bay K 86444 was evaluated by tensions at initial (3 min after the addition) and plateau (20 min after the addition) phases. Relative vasomotor response (contraction/relaxation potential) of a given ring was evaluated by the ratio of reduced tension (g) by ACh to increased tension (g) by PE.

Relaxation experiments evaluating the VDCC blockers (nifedipine, verapamil and Trp-His) were performed on 1 µM PE-contracted rings. Once the PE-contracted tension plateaued, each blocker was individually added to the bath to assess relaxation activity in a cumulative manner (nifedipine; 0.001–1.7 µM, verapamil; 0.01–50 µM, Trp-His; 0.1–5.8 mM). Since the time schedule for each addition was within an ∼10 min interval and was consistent in each set of experiments, the bias in the cumulative experiments would be negligible. Relaxation activity was evaluated as the EC_50_ value, the effective concentration producing 50% relaxation of PE-induced contracted tension.

### Western Blot Analysis

The amounts of AT1/2R and alpha-1c subunit of Cav1.2 VDCC were detected by Western blot analysis. Each thoracic aorta isolated from both 8- and 40-week WKY and SHR was homogenized with a radio-immunoprecipitation assay (RIPA) buffer (150 mM NaCl, 0.5% sodium deoxycholate, 0.1% sodium sulfate, 1.0% Nonidet P-40, and 50 mM Tris-HCl, pH 8.0) containing 1 mM phenylmethylsulfonyl fluoride on ice. Then, the homogenate was subjected to sonication 10 times for 10 sec each on ice, followed by centrifugation at 14,000 *g* for 15 min at 4°C. The protein concentration was determined with a Bio-Rad DC Protein Assay Kit (bovine serum albumin (BSA) as a standard). The extract was mixed with an equal volume of sample buffer (20% glycerol, 4% SDS, 3% dithiothreitol, 0.002% bromophenol blue and 0.125 M Tris-HCl, pH 6.8) and maintained at 100°C for 10 min. An aliquot (20 µg protein/lane) was applied to either a 10 or 12% SDS-PAGE gel for 1.5 h at 20 mA and transferred onto a polyvinylidene fluoride membrane (Hybond-P, GE Healthcare) for 1.5 h at 40 mA. The membrane was blocked for 1 h at room temperature with 5% ECL blocking agent (GE Healthcare) in TBS-Tween20 (TBS-T, 20 mM Tris-HCl, 137 mM NaCl and 0.05% Tween-20, pH 7.6). The membrane was probed with primary antibodies to either AT1R, AT2R, alpha-1c subunit of Cav1.2 VDCC (1∶2000 dilution) or β-actin (1∶1000 dilution) overnight at 4°C and then incubated with the secondary antibody (anti-rabbit for AT1/2R and for VDCC or anti-mouse for β-actin, 1∶1000 dilution) for 1 h at room temperature. The membrane was analyzed by ECL detection reagents with an Image Quant LAS 4000 (GE Healthcare). Quantification of the amount of target protein was determined by an Image Quant TL 7.0 software (GE Healthcare). The level of each protein (AT1/2R and alpha-1c subunit of Cav1.2 VDCC) was calculated by the ratio of the levels of each target protein to β-actin, and the ratio of each target protein level in 8-week WKY was denoted as 1.0.

### Confocal Microscopy

Immunohistochemical analysis of target proteins in the thoracic artery was performed, according to the method used by Idris-Khodja and Schini-Kerth [14]. Briefly, segments of thoracic artery were removed and snap-frozen in liquid nitrogen. Frozen aorta was sliced into 14-µm-thick sections at the cross-sectional face using a CM1100 Leica Cryomicrotome (Leica, Wetzler, Germany). The sections were fixed with 4% paraformaldehyde, followed by blocking treatment with 5% BSA in phosphate buffered saline (PBS) containing 0.1% Triton X-100 for 1 h at room temperature. The sections were then incubated with antibodies to AT1R (1∶200 dilution), AT2R (1∶100 dilution) and alpha-1c subunit of Cav1.2 VDCC (1∶100 dilution) overnight at 4°C, following incubation with secondary antibody (Alexa 488-conjugated goat anti-rabbit IgG, 1∶400 dilution) for 2 h at room temperature in the dark. Target proteins on the sections were visualized using a Nikon confocal microscope (Nikon, Tokyo, Japan).

### Statistical Analyses

Results are expressed as the mean ± standard error of the mean (SEM). Statistical differences between groups were evaluated by one-way ANOVA, followed by the Tukey-Kramer's *t*-test for *post hoc* analysis. *P*-values less than 0.05 were considered to be significant. All analyses were performed with a Stat View J 5.0 software (SAS Institute Inc., Cary, NC, USA).

## Results

### Vasomotor Response in Both Young and Aged WKY and SHR

**Figure 1:**
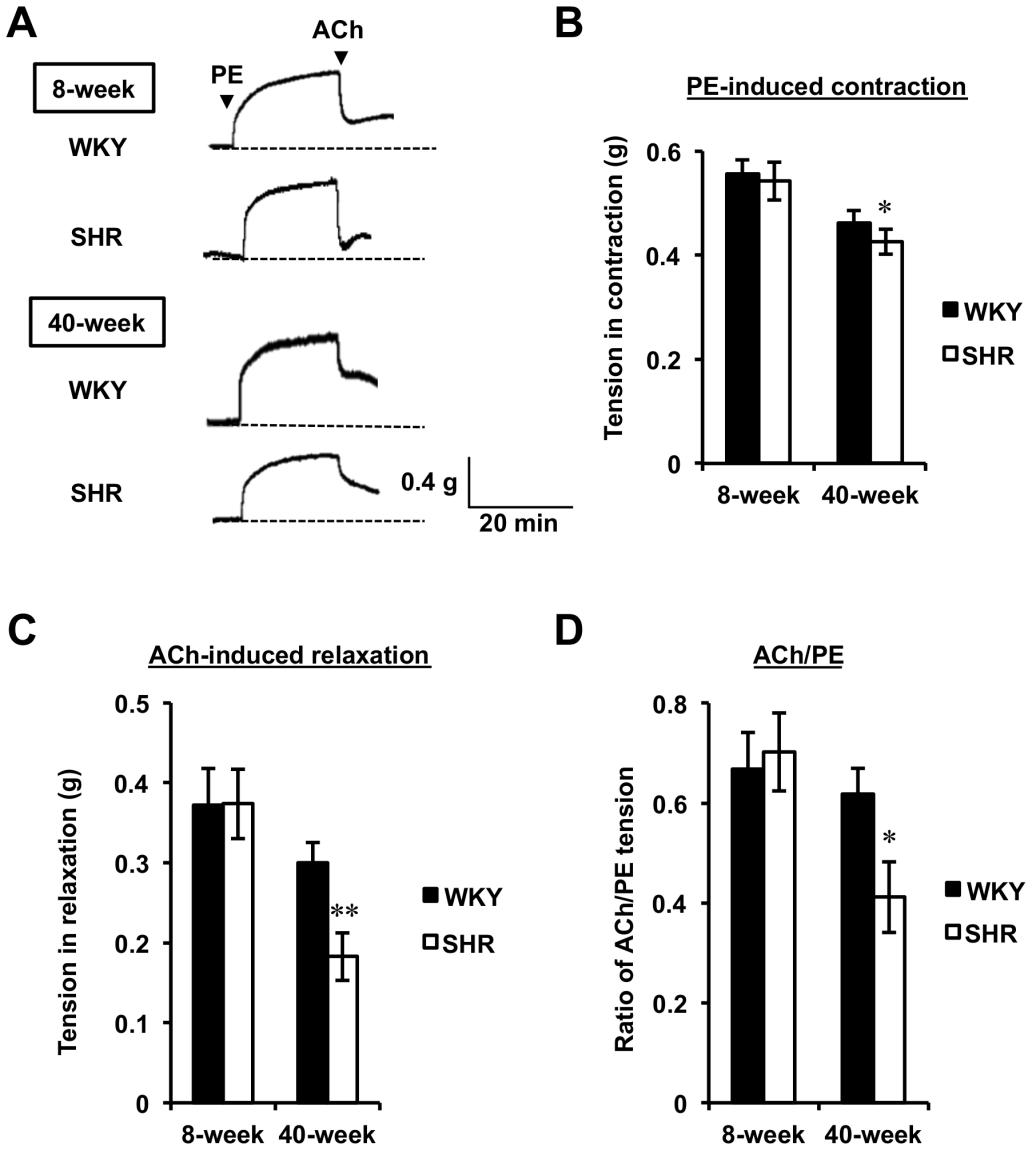
Effect of Age and Rat Strain on Vasomotor Responses in Thoracic Aortic Rings. Representative traces (A) were recorded in PE (1 µM)-induced contraction and the subsequent ACh (10 µM)-induced relaxation of aortic rings. Tensions in contraction (B) and relaxation (C) were measured for aortic rings taken from both 8- and 40-week WKY and SHR. The ratio of reduced tension by ACh to increased tension by PE was used for the vasomotor response index (D). Results are expressed as the mean ± SEM values (n = 4–8). ^*^ *P*<0.05, ^**^ *P*<0.01 vs 8-week SHR.

The maximum contraction/relaxation response of the aorta against vaso-agonists with aging was examined in 1 µM PE- and the subsequently 10 µM ACh-stimulated aortic rings from both 8- or 40-week WKY and SHR (Figure 1A). As shown in Figure 1B, the PE-induced contraction response was not influenced by rat strain at the young (8-week old) stage (tension: WKY, 0.56±0.03 g; SHR, 0.54±0.04), whereas at the aged (40-week old) stage a significant (*P*<0.05) reduction in the contraction response was observed only for SHR (tension, 0.43±0.02 g). Taking into account for the tendency of reduced contraction for 40-week WKY (tension: 0.46±0.02 g), aging may affect PE-induced contraction of the aorta rather than hypertensive disease. We also found a similar spectrum for ACh-induced relaxation (Figure 1C) with the contraction spectrum obtained in Figure 1B; a significant reduction in the relaxation response by 10 µM ACh was observed only for the 40-week SHR, while no significant changes in the relaxation response were observed among the other groups. Taking together these results and the resulting vasomotor profile (ratio of tension by ACh to tension by PE, Figure 1D), the maximum contraction/relaxation response of rat aorta induced by PE/ACh was greatly affected or reduced in aged rats, whereas impaired vessel response in hypertension was less or negligible within our rat groups.

### Ang II-Induced Contraction in Both WKY and SHR

**Figure 2:**
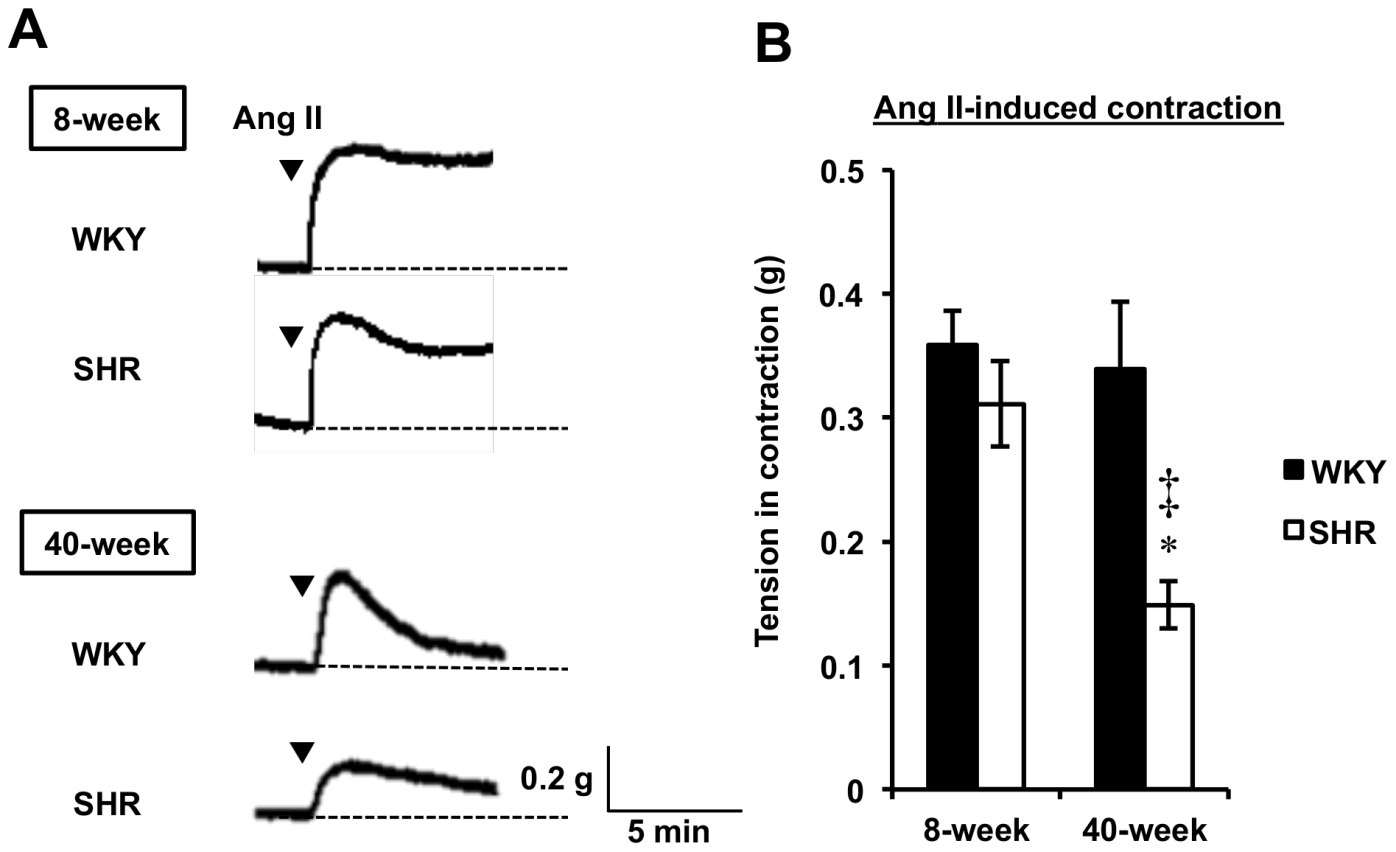
Profiles of Ang II-Induced Contraction in Both WKY and SHR. Representative traces (A) were recorded in Ang II (1 µM)-induced contraction of aortic rings. Tension in contraction by Ang II was measured in aortic rings from both 8- and 40-week WKY and SHR in the presence of PD123177 (1 µM, AT2R inhibitor) (B). Results are expressed as the mean ± SEM (n = 4–5). ^*^ *P*<0.05 vs 8-week SHR, ^‡^ *P*<0.01 vs 40-week WKY.

Ang II (1 µM), a contraction agonist, was used to evaluate the contraction response through the activation of the ATR-related signaling pathway in aortic rings from both 8- and 40-week WKY and SHR (Figure 2). As shown in Figure 2A, Ang II stimulation evoked a rapid contraction as well as a sustained contraction response for each ring, except rings from both 40-week WKY and SHR rats. In addition, aortic rings from the 40-week SHR significantly (*P*<0.05 vs 8-week SHR and *P*<0.01 vs 40-week WKY) attenuated the maximal contraction response induced by Ang II, unlike rings from the other groups (8-week SHR, 0.31±0.03 g; 40-week SHR, 0.15±0.02 g) (Figure 2B). This finding indicated that hypertension may influence the contraction responsiveness to Ang II stimulation in aged SHRs.

### Effect of Age on Vascular AT1R and AT2R Expressions in Both WKY and SHR

**Figure 3:**
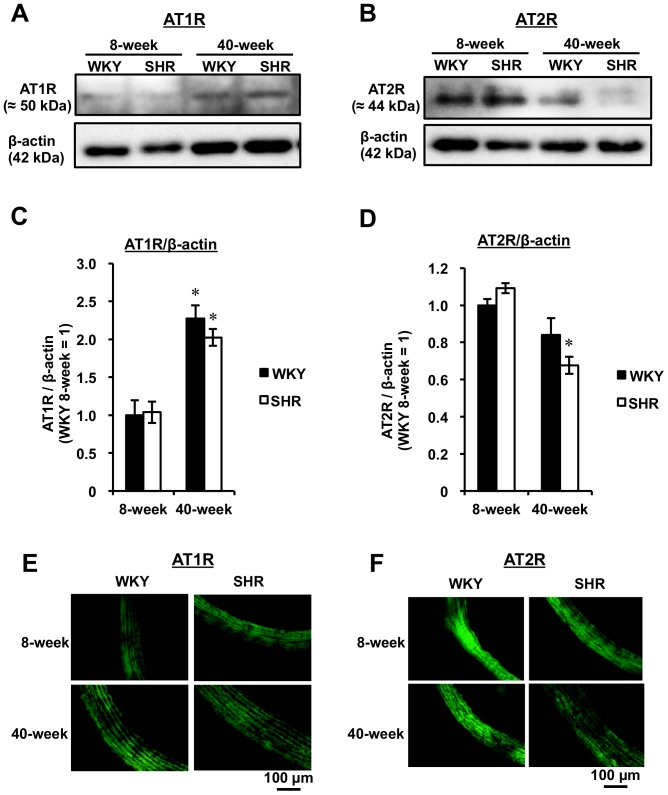
Protein Expressions of AT1R and AT2R in Aorta by Western Blot Analysis and Confocal Microscopy. Representative blots are shown for AT1R (A) and AT2R (B). The amounts of AT1R and AT2R were determined as the ratio of either AT1R (C) or AT2R (D) to β-actin. Results are expressed as the mean ± SEM (n = 3–5). ^*^ *P*<0.05 vs each 8-week rat strain. The expression levels were determined by immunofluorescence staining. Confocal measurements of both AT1R (E) and AT2R (F) were performed on rat aortic segments (14 µm).

The protein levels of AT1R and the AT2R were evaluated in thoracic aortae from both 8- and 40-week WKY and SHR. As shown in Figures 3A and C, AT1R protein expression was markedly elevated (*P*<0.05) with age for both rat strains. In contrast, a reduced AT2R protein expression level with age was observed, in particular for the 40-week SHR, in which the AT2R level was markedly decreased (Figures 3B and D). Evaluation of each receptor expression level in thoracic aortic sections from each rat by confocal microscopy also showed that aging induced an enhanced AT1R expression and a reduced AT2R expression (Figures 3E and F).

### Bay K 8644-Induced Contraction in Both WKY and SHR

**Figure 4:**
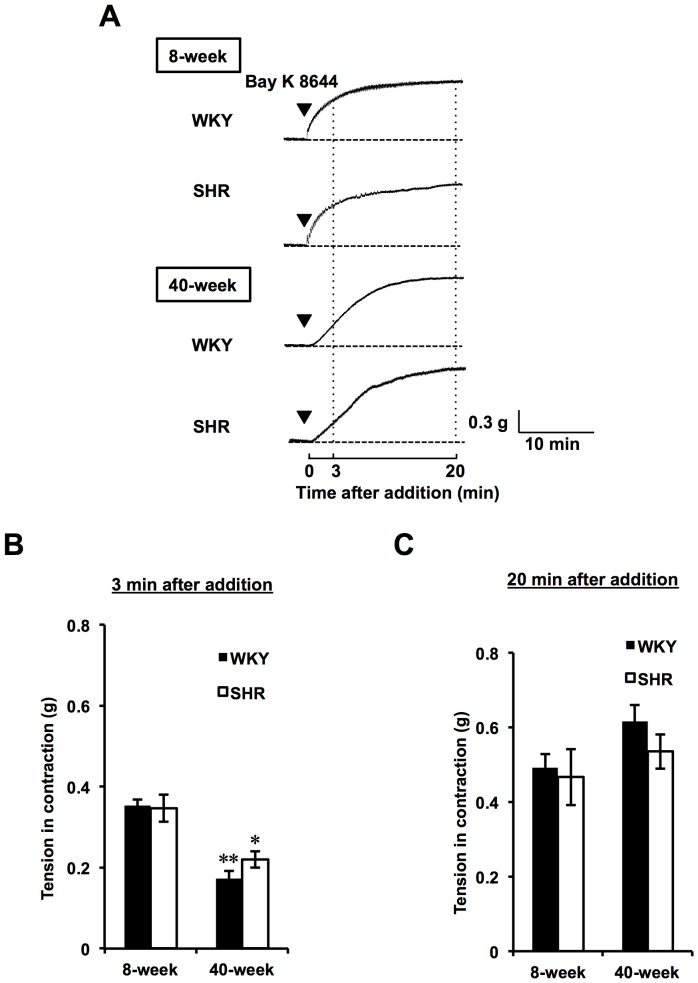
Profiles of Bay K 8644-induced contraction in Both WKY and SHR. Representative traces (A) were recorded in Bay K 8644 (1 µM)-induced contracted aortic rings. Tension in contraction by Bay K 8644 was measured in aortic rings from both 8- and 40-week WKY and SHR at initial (3 min after the addition) phase (B) and at plateau (20 min after the addition) phase (C). Results are expressed as the mean ± SEM (n = 3). ^*^ *P*<0.05, ^**^ *P*<0.01 vs each 8-week rat strain.

We next evaluated the effect of Bay K 8644 (1 µM) a VDCC agonist, on the contraction response of rings from age-matched WKY and SHR at initial and plateau contraction phases. As shown in Figure 4A, a slower contraction responsiveness to Bay K 8644 was observed at initial phase for both 40-week rats compared to 8-week rats, although contraction tensions at plateau phase for 40-week rats were comparable for those for 8-week rats. Namely, the activity of 1 µM Bay K 8644 for contraction at 3 min after the addition was significantly reduced in both 40-week rat groups compared to those in the 8-week groups (8-week WKY, 0.35±0.02 g; 8-week SHR, 0.35±0.04 g; 40-week WKY, 0.17±0.02 g; 40-week SHR, 0.22±0.02 g) (Figure 4B), suggesting that an initial extracellular Ca^2+^ incorporation via VDCC by Bay K 8644 stimulation was disrupted with age, irrespective of rat strain.

### Effect of Age on Vascular VDCC Expression in Both WKY and SHR

**Figure 5:**
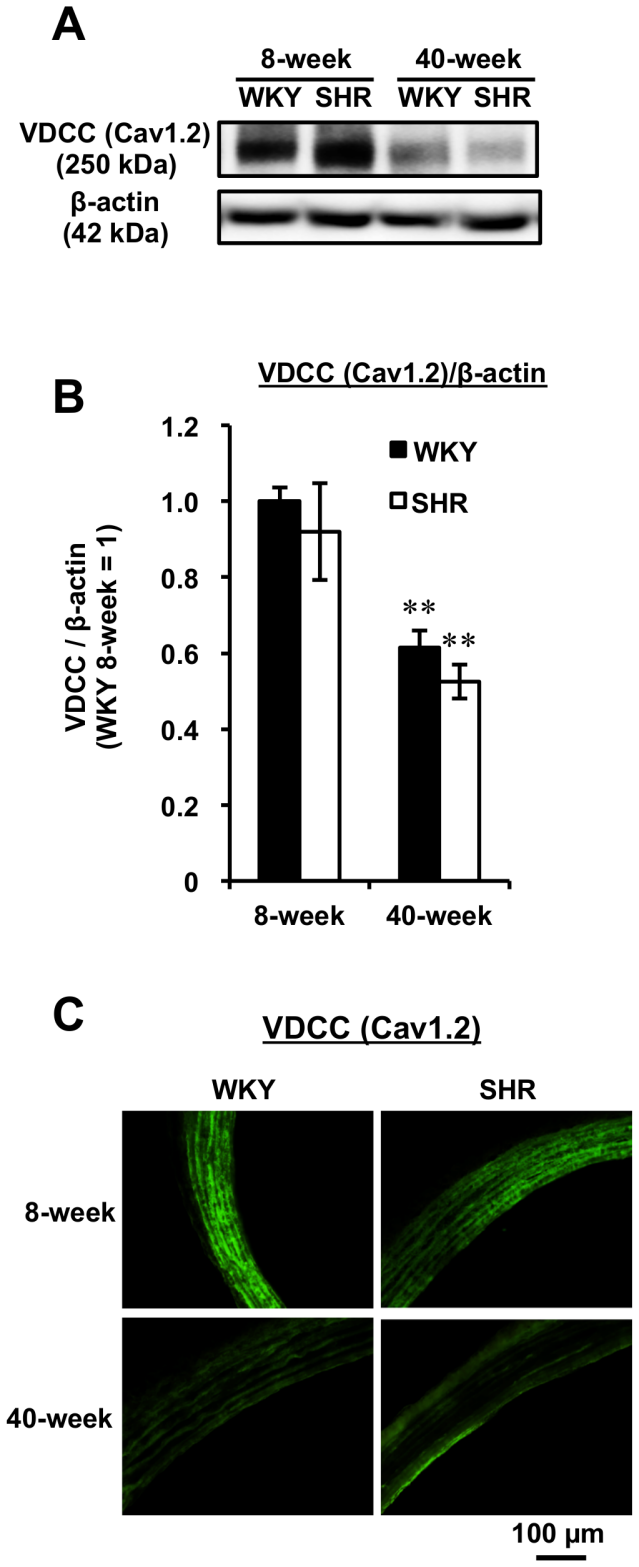
Protein Expression of VDCC in Aorta by Both Western Blot Analysis and Confocal Microscopy. Representative blots are shown for VDCC (A). The amount of the alpha-1c subunit of VDCC was determined as the ratio of VDCC to β-actin (B). Results are expressed as the mean ± SEM (n = 6). ^**^ *P*<0.01 vs each 8-week rat strain. Confocal measurement of VDCC (C) was performed in rat aortic segments (14 µm).

To account for the blunted Bay K 8644-induced contraction response with age (Figure 4), the expression of the alpha-1c subunit of Cav1.2 VDCC was assessed by both Western blot analysis and confocal microscopy. As shown in Figure 5A, VDCC protein was significantly reduced with age for both rat strains, and no significant differences between age-matched rats were observed (Figure 5B). A marked difference in VDCC protein expression between 8- and 40-week rats was also observed in immuno-stained aortic sections by confocal microscopy (Figure 5C). These findings raised the possibility that aging attenuated VDCC expression to suppress extracellular Ca^2+^ entry into rat vessels.

### Effect of Age on Relaxation Activity of VDCC Blockers

**Figure 6:**
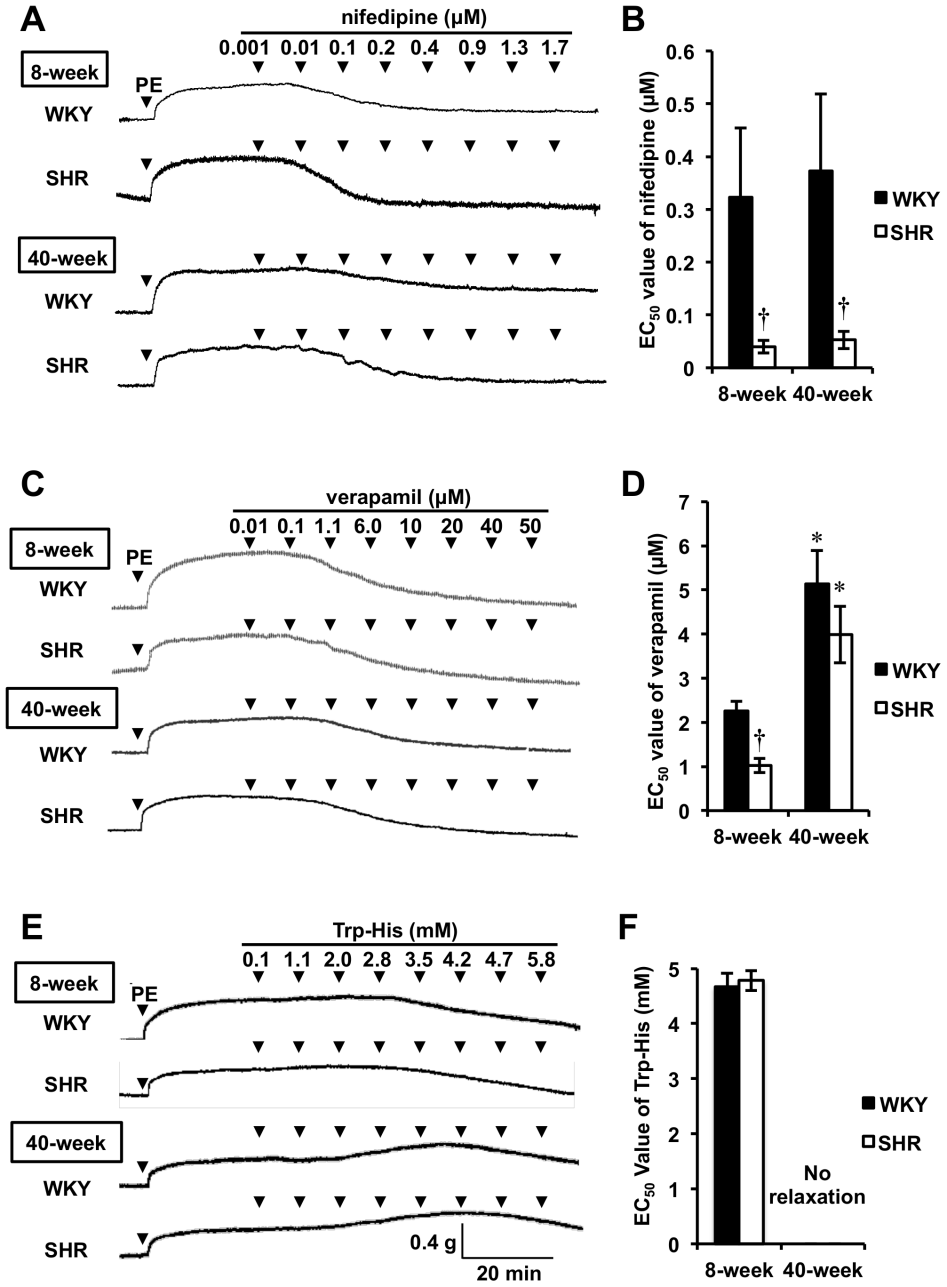
Profiles of Relaxation by VDCC Blockers in Both WKY and SHR. Representative traces of nifedipine (A)-, verapamil (C)-, and Trp-His (E)-induced relaxation in PE (1 µM)-contracted aortic rings from both 8- and 40-week WKY and SHR. The relaxation activity was represented as EC_50_ values for nifedipine (B), verapamil (D), and Trp-His (F). Results are expressed as the mean ± SEM values (n = 3–7). ^*^ *P*<0.05 vs each 8-week rat strain, ^†^ *P*<0.05 vs age-matched WKY.

Since we found that VDCC protein expression was attenuated with age in rats, we next investigated whether therapeutic and natural VDCC blockers could affect relaxation activity in aged rats. Nifedipine (0.001–1.7 µM) and verapamil (0.01–50 µM) as well as Trp-His (0.1–5.8 mM) were used as blockers for 1 µM PE-induced contraction. As shown in Figures 6C–F, verapamil and Trp-His (a natural VDCC blocker) significantly lost their relaxation activity. Surprisingly, Trp-His, a natural preventive compound for vascular diseases [11], was inactive in aged rats. In contrast, nifedipine still possessed relaxation activity in SHR, but not in WKY, despite aging (EC_50_: 8-week WKY, 0.32±0.13 µM; 8-week SHR, 0.04±0.01 µM; 40-week WKY, 0.37±0.15 µM; 40-week SHR, 0.05±0.02 µM, Figures 6A and B).

## Discussion

In this study, we investigated whether aging in rats influences vasomotor tone using thoracic aortic rings from both young (8-week) and aged (40-week) WKY and SHR. In contraction experiments using aortic rings exposed to PE and to Ang II, it was found that aging in SHR greatly attenuated their contraction potentials, while WKY retained the response, irrespective of the age of the rats. A significant influence of age on the vascular response was observed in a Bay K 8644 contraction study, in which contraction induced by Bay K 8644 (a VDCC agonist) was significantly lower in both aged WKY and SHR than that in young rats. In addition, we demonstrated for the first time that aortic VDCC expression in aged rats was much lower than that in young rats, irrespective of the presence of hypertension. The finding that VDCC blockers (e.g., verapamil and Trp-His) lost their relaxation activity in 40-week rats also suggests that their usefulness as therapeutic treatments in aged patients may be limited and needs to be evaluated.

So far, some researchers have investigated the effect of age on vascular function from the view-points of prevention or treatment of cardiovascular and cerebral vascular disorders. Van der Loo et al. [15] reported that aging promoted peroxynitrite formation by increased superoxide anion formation in the vascular endothelium in F344/BN F1 rats, and speculated on the importance of suppression of oxidative stress for age-related vascular dysfunction. Factors affecting age-related endothelial dysfunction were also reported to involve ATPases [16] and NADPH oxidases [17]. Owing to these findings, some preventive studies against age-induced vascular dysfunction have been performed to improve endothelium-dependent vascular relaxation by antioxidant compounds, e.g., thymoquinone [14], red wine polyphenols [18], and vitamin C [19].

Research interests in age-related vascular dysfunction have begun to investigate the change in the physiological vascular response in the muscle layer, since vasomotor activity is regulated by MLC phosphorylation through in part AT1R stimulation. It is well known that the blockade of AT1R by AT1R antagonistic drugs (e.g., losartan) is the most effective target for therapeutics for hypertension. Age-related studies on AT1R expression demonstrate that it was enhanced with age in SHR [4] and in Sprague-Dawley rats [20]. In contrast, AT2R expression, which involves the activation of relaxation-signaling cascades [21], was attenuated with age [4]. The contradictory expression patterns of both receptors with age do not fully explain the underlying mechanism of vasomotor response with age. In this study, we demonstrated similar findings in WKY and in SHR as already reported (Figure 3), indicating that hypertension did not affect the ATR expression patterns with age. A marked reduction of Ang II-induced contraction in only 40-week SHR aortic rings with blocking of the AT2R-related signaling pathways (Figure 2) provided further information that reduced AT1R-contraction in aged SHR would result from any reduction of AT1R-related signaling cascade by hypertension regardless of enhanced AT1R expression with age. A similar spectrum was also obtained in aged Fisher 344 rats [22] and in SHR [23]. Alternatively, as Arun et al. reported [24], altered [Ca^2+^]_i_ incorporation through reduction in the AT1R-coupled VDCC phosphorylation pathway may be involved in the reduced contraction by Ang II in aged SHR. Contrary to this finding, as Davare and Hell [25] revealed that aging caused increased phosphorylation of VDCC in neuronal network system of the brains. Thus, further experiments regarding VDCC phosphorylation in rats are needed to clarify whether aging alters Ca^2+^ sensitivity via aortic VDCC.

In the present study, we demonstrated that aging reduced contraction responsiveness at initial phase to VDCC agonist, Bay K 8644, for both WKY and SHR, although tensions at plateau contraction phase were unaltered between young and aged rats as similar to the result by Hernandez et al. [26] (Figure 4). The slower responsiveness to Bay K 8644 at initial contraction phase in aged rats (Figure 4A) would be caused by reduced activation of VDCC [26] or reduced VDCC expression with age. In this study, we demonstrated that aging reduced the aortic L-type VDCC protein expression in rats (Figure 5) for the first time. Similar findings have been reported for sinoatrial node [5] and for cerebral artery [6]. However, it still remains unclear whether aging may affect the expression of other Ca^2+^ channel subtypes such as R- and T-type, located in the heart and in the kidney [27]. As speculated from the reduced VDCC expression in aged rats, the attenuated relaxation activity of VDCC blockers (verapamil [28] and Trp-His [9]) occurred in PE-contraction aortic rings (Figure 6). In contrast, no change in the potent relaxation activity of nifedipine, another type of VDCC blocker, between young and aged SHR (and WKY) was found irrespective to their different VDCC expressions (Figures 5 and 6). This might be explained by alternative roles of nifedipine in vasorelaxation signaling pathways by activating AMP-activated protein kinase [29] and endothelial NO synthase [30]. However, further study on the relationship between activation of relaxation-signaling pathways by VDCC blockers and aging is needed. The disappearance of the relaxation potential of Trp-His in aged rats also provided useful information suggesting that bioactive natural compounds having preventive health-benefits for vascular disease via VDCC blocking action [9], [10] may fail to evoke the preventive potential with aging.

In conclusion, we have clarified that aging greatly attenuated the PE- and Ang II-induced contractions in SHR, whereas WKY did not loss the contraction responses. Together with the results on enhanced AT1R and reduced AT2R in both aged WKY and SHR, we demonstrated for the first time that L-type VDCC protein expression was greatly reduced with age in rats. The reduced VDCC with age caused the disappearance of the relaxation potential of VDCC blockers. The present study will, thus, form the basis for future approaches for the prevention of vascular diseases at age.

## References

[1] Moore A, Mangoni AA, Lyons D, Jackson SHD (2003) The cardiovascular system. Br J Clin Pharmacol 56: 254–260. 12919173 10.1046/j.0306-5251.2003.01876.x PMC1884359

[2] Herrera MD, Mingorance C, Rodríguez-Rodríguez R, Alvarez de Sotomayor M (2010) Endothelial dysfunction and aging: an update. Ageing Res Rev 9: 142–152. 19619671 10.1016/j.arr.2009.07.002

[3] Yildiz O (2007) Vascular smooth muscle and endothelial functions in aging. Ann N Y Acad Sci 1100: 353–360. 17460198 10.1196/annals.1395.038

[4] Touyz RM, Endemann D, He G, Li J-S, Schiffrin EL (1999) Role of AT2 Receptors in Angiotensin II Stimulated Contraction of Small Mesenteric Arteries in Young SHR. Hypertension 33: 366–372. 9931131 10.1161/01.hyp.33.1.366

[5] Jones SA, Boyett MR, Lancaster MK (2007) Declining into failure: the age-dependent loss of the L-type calcium channel within the sinoatrial node. Circulation 115: 1183–1190. 17339548 10.1161/CIRCULATIONAHA.106.663070

[6] Georgeon-Chartier C, Menguy C, Prévot A, Morel J-L (2013) Effect of aging on calcium signaling in C57Bl6J mouse cerebral arteries. Pflugers Arch 465: 829–838. 23238969 10.1007/s00424-012-1195-7

[7] Somlyo AP, Somlyo A V (1994) Signal transduction and regulation in smooth muscle. Nature 372: 231–236. 7969467 10.1038/372231a0

[8] Ishii N, Matsumura T, Kinoshita H, Fukuda K, Motoshima H, et al (2010) Nifedipine induces peroxisome proliferator-activated receptor-gamma activation in macrophages and suppresses the progression of atherosclerosis in apolipoprotein E-deficient mice. Arterioscler Thromb Vasc Biol 30: 1598–1605. 20508203 10.1161/ATVBAHA.109.202309

[9] Wang Z, Watanabe S, Kobayashi Y, Tanaka M, Matsui T (2010) Trp-His, a vasorelaxant di-peptide, can inhibit extracellular Ca^2+^ entry to rat vascular smooth muscle cells through blockade of dihydropyridine-like L-type Ca^2+^ channels. Peptides 31: 2060–2066. 20688122 10.1016/j.peptides.2010.07.013

[10] Kobayashi Y, Fukuda T, Tanaka M, Matsui T (2012) The anti-atherosclerotic di-peptide, Trp-His, inhibits the phosphorylation of voltage-dependent L-type Ca^2+^ channels in rat vascular smooth muscle cells. FEBS Open Bio 2: 83–88. 10.1016/j.fob.2012.04.005 PMC3642122 23650584

[11] Matsui T, Sato M, Tanaka M, Yamada Y, Watanabe S, et al (2010) Vasodilating dipeptide Trp-His can prevent atherosclerosis in apo E-deficient mice. Br J Nutr 103: 309–313. 19728894 10.1017/S0007114509991814

[12] Tanaka M, Zhao J, Suyama A, Matsui T (2012) Epigallocatechin gallate promotes the vasorelaxation power of the antiatherosclerotic dipeptide Trp-His in contracted rat aorta. J Agric Food Chem 60: 9048–9054. 22900606 10.1021/jf3010228

[13] Timmermans PB, Wong PC, Chiu AT, Herblin WF, Benfield P, et al (1993) Angiotensin II receptors and angiotensin II receptor antagonists. Pharmacol Rev 45: 205–251. 8372104

[14] Idris-Khodja N, Schini-Kerth V (2012) Thymoquinone improves aging-related endothelial dysfunction in the rat mesenteric artery. Naunyn Schmiedebergs Arch Pharmacol 385: 749–758. 22526469 10.1007/s00210-012-0749-8

[15] Van der Loo B, Labugger R, Skepper JN, Bachschmid M, Kilo J, et al (2000) Enhanced peroxynitrite formation is associated with vascular aging. J Exp Med 192: 1731–1744. 11120770 10.1084/jem.192.12.1731 PMC2213492

[16] Büssemaker E, Popp R, Fisslthaler B, Larson CM, Fleming I, et al (2003) Aged spontaneously hypertensive rats exhibit a selective loss of EDHF-mediated relaxation in the renal artery. Hypertension 42: 562–568. 12925561 10.1161/01.HYP.0000088852.28814.E2

[17] Wind S, Beuerlein K, Armitage ME, Taye A, Kumar AHS, et al (2010) Oxidative stress and endothelial dysfunction in aortas of aged spontaneously hypertensive rats by NOX1/2 is reversed by NADPH oxidase inhibition. Hypertension 56: 490–497. 20606112 10.1161/HYPERTENSIONAHA.109.149187

[18] Mudnic I, Modun D, Rastija V, Vukovic J, Brizic I, et al (2010) Antioxidative and vasodilatory effects of phenolic acids in wine. Food Chem 119: 1205–1210.

[19] Taddei S, Virdis A, Ghiadoni L, Magagna A, Salvetti A (1998) Vitamin C Improves Endothelium-Dependent Vasodilation by Restoring Nitric Oxide Activity in Essential Hypertension. Circulation 97: 2222–2229. 9631871 10.1161/01.cir.97.22.2222

[20] Schulman IH, Zhou M-S, Treuer A V, Chadipiralla K, Hare JM, et al (2010) Altered renal expression of angiotensin II receptors, renin receptor, and ACE-2 precede the development of renal fibrosis in aging rats. Am J Nephrol 32: 249–261. 20689271 10.1159/000318607

[21] Widdop RE (2002) AT2 Receptor-Mediated Relaxation Is Preserved After Long-Term AT1 Receptor Blockade. Hypertension 40: 516–520. 12364356 10.1161/01.hyp.0000033224.99806.8a

[22] Cai G, Gurdal H, Seasholtz TM, Johnson MD (1994) Age-related changes in angiotensin II-stimulated vascular contraction and inositol phosphate accumulation in Fischer 344 rats. Mech Ageing Dev 76: 125–133. 7885059 10.1016/0047-6374(94)91587-3

[23] Endemann D, Touyz RM, Li JS, Deng LY, Schiffrin EL (1999) Altered angiotensin II-induced small artery contraction during the development of hypertension in spontaneously hypertensive rats. Am J Hypertens 12: 716–723. 10411369 10.1016/s0895-7061(99)00036-9

[24] Arun KHS, Kaul CL, Ramarao P (2005) AT1 receptors and L-type calcium channels: functional coupling in supersensitivity to angiotensin II in diabetic rats. Cardiovasc Res 65: 374–386. 15639476 10.1016/j.cardiores.2004.10.010

[25] Davare M a, Hell JW (2003) Increased phosphorylation of the neuronal L-type Ca(2+) channel Ca(v)1.2 during aging. Proc Natl Acad Sci U S A 100: 16018–16023. 14665691 10.1073/pnas.2236970100 PMC307685

[26] Hernández MC, Salaices M, Ponte A, Alonso MJ, Sánchez-Ferrer CF, et al (1995) Effects of Bay K 8644 in aorta from spontaneously hypertensive and Wistar Kyoto rats of different ages. J Auton Pharmacol 15: 257–269. 8576273 10.1111/j.1474-8673.1995.tb00309.x

[27] Hayashi K, Wakino S, Sugano N, Ozawa Y, Homma K, et al (2007) Ca2+ channel subtypes and pharmacology in the kidney. Circ Res 100: 342–353. 17307972 10.1161/01.RES.0000256155.31133.49

[28] Godfraind T (1994) Calcium antagonists and vasodilatation. Pharmacol Ther 64: 37–75. 7846116 10.1016/0163-7258(94)90033-7

[29] Sung JY, Choi HC (2011) Nifedipine inhibits vascular smooth muscle cell proliferation and reactive oxygen species production through AMP-activated protein kinase signaling pathway. Vascul Pharmacol 56: 1–8. 21708289 10.1016/j.vph.2011.06.001

[30] Ding Y, Vaziri ND (2000) Nifedipine and diltiazem but not verapamil up-regulate endothelial nitric-oxide synthase expression. J Pharmacol Exp Ther 292: 606–609. 10640297

